# Erratum to: 18β-Glycyrrhetinic acid exerts protective effects against cyclophosphamide-induced hepatotoxicity: potential role of PPARγ and Nrf2 upregulation

**DOI:** 10.1186/s12263-016-0548-9

**Published:** 2016-11-23

**Authors:** Ayman M. Mahmoud, Hussein S. Al Dera

**Affiliations:** 1 Physiology Division, Zoology Department, Faculty of Science, Beni-Suef University, Beni Suef, 62514 Egypt; 2 Basic Medical Sciences Department, King Saud bin Abdulaziz University for Health Sciences, Riyadh, Saudi Arabia

## Erratum

In the original version of this article [1], the used housekeeping gene was reported incorrectly as GAPDH in the Methods section. It should be β-actin, as described in Table 1 in the published article.
